# The Basic Helix-Loop-Helix Transcription Factor SmbHLH1 Represses Anthocyanin Biosynthesis in Eggplant

**DOI:** 10.3389/fpls.2021.757936

**Published:** 2021-11-12

**Authors:** Zhaofei Duan, Shiyu Tian, Guobin Yang, Min Wei, Jing Li, Fengjuan Yang

**Affiliations:** ^1^State Key Laboratory of Crop Biology, College of Horticulture Science and Engineering, Shandong Agricultural University, Shandong, China; ^2^Scientific Observing and Experimental Station of Facility Agricultural Engineering (Huang-Huai-Hai Region), Ministry of Agriculture and Rural Affairs, Shandong, China; ^3^Shandong Collaborative Innovation Center for Fruit and Vegetable Production With High Quality and Efficiency, Tai’an, China; ^4^Key Laboratory of Biology and Genetic Improvement of Horticultural Crops in Huanghuai Region, Ministry of Agriculture and Rural Affairs, Shandong, China

**Keywords:** eggplant, anthocyanin biosynthesis, bHLH transcription factor, negative regulation, RNA-seq

## Abstract

Many basic helix-loop-helix transcription factors (TFs) have been reported to promote anthocyanin biosynthesis in numerous plant species, but little is known about bHLH TFs that inhibit anthocyanin accumulation. In this study, SmbHLH1 from *Solanum melongena* was identified as a negative regulator of anthocyanin biosynthesis. However, SmbHLH1 showed high identity with SmTT8, which acts as a SmMYB113-dependent positive regulator of anthocyanin-biosynthesis in plants. Overexpression of *SmbHLH1* in eggplant caused a dramatic decrease in anthocyanin accumulation. Only the amino acid sequences at the N and C termini of SmbHLH1 differed from the SmTT8 sequence. Expression analysis revealed that the expression pattern of *SmbHLH1* was opposite to that of anthocyanin accumulation. Yeast two-hybrid (Y2H) and bimolecular fluorescence complementation (BiFC) assays showed that SmbHLH1 could not interact with SmMYB113. Dual-luciferase assay demonstrated that SmbHLH1 directly repressed the expression of *SmDFR* and *SmANS*. Our results demonstrate that the biological function of *bHLH*s in anthocyanin biosynthesis may have evolved and provide new insight into the molecular functions of orthologous genes from different plant species.

## Introduction

Anthocyanins are water-soluble pigments that are responsible for the red, purple, and blue colors of different organs in a wide range of plants (Mol et al., 1998; Koes et al., 2005; Allan et al., 2008). In addition to coloring flowers and fruits to attract pollinators and seed dispersers, anthocyanins can also protect plants from UV damage and promote resistance to low-temperature stress in plants (Ilk et al., 2015). Anthocyanins have been found to be the most powerful free radical scavengers in plants, and they have been used to lower blood pressure, improve vision, reduce inflammation, and prevent cancer (Konczak and Zhang, 2004; Tsuda, 2012).

Anthocyanins are an important branch of the flavonoid biosynthetic pathway and are formed in a stepwise series of enzymatic reactions catalyzed by chalcone synthase (CHS), chalcone isomerase (CHI), flavanone-3-hydroxylase (F3H), flavonoid 3′-hydroxylase (F3′H), flavonoid 3′5′-hydroxylase (F3′5′H), dihydroflavonol 4-reductase (DFR), anthocyanidin synthase (ANS), and a 3-glucosyltransferase (3GT; Koes et al., 2005). These anthocyanin biosynthetic genes are transcriptionally regulated by a MYB-bHLH-WD40 (MBW) complex, the crucial role of which has been well-documented across many plants, such as *Arabidopsis*, apple, tomato, and so on (Xie et al., 2012; Tohge et al., 2017; Sun et al., 2020a). AcMYB123 and AcbHLH42, the *Actinidia chinensis* cv. Hongyang orthologs of *Arabidopsis* TT2 (MYB) and TT8 (bHLH), respectively, interact to activate the promoters of *AcANS* and *AcF3GT1* and elevate anthocyanin accumulation in *Actinidia arguta* cv. Baby star and transgenic *Arabidopsis thaliana* (Wang et al., 2019). In apple, MdbHLH3 interacts with MdMYB1, MdMYB9, and MdMYB11 to promote anthocyanin or proanthocyanidin (PA) accumulation (Xie et al., 2012; An et al., 2014).

The bHLH proteins are the second largest class of transcription factors (TFs) in plants; they can be divided into 26 subgroups (Pires and Dolan, 2010), and this classification can be extended to 32 subgroups if the “atypical” bHLHs are included (Carretero-Paulet et al., 2010). Compared with the 11–13 subgroups of the R2R3 MYBs (Li et al., 2012; He et al., 2016; Huang et al., 2018), bHLH family seems to have a more complex role in anthocyanin regulation. In general, the first 200 amino acids of the bHLH protein are involved in the interaction with the MYB partner, whereas the following 200 amino acids interact with the WD40 protein (Montefiori et al., 2015).

The G-box (5′-CACGTG-3′) or E-box (5′-CANNTG-3′) in gene promoter regions is recognized by bHLH family members (Atchley et al., 1999; Montefiori et al., 2015; Wang et al., 2018). Strikingly, the promoters of most flavonoid or anthocyanin biosynthetic genes contain G-box and E-box elements (Massari and Murre, 2000). Therefore, in addition to forming the MBW complex, bHLHs can also directly regulate anthocyanin biosynthesis. For example, MdbHLH3 can activate the expression of *MdMYB1*, *MdMYB9*, and *MdMYB11* to promote anthocyanin accumulation (Xie et al., 2012; An et al., 2014). TT8 is known to interact with MYB protein to enhance anthocyanin biosynthesis (Baudry et al., 2006; Li et al., 2016b; Wang et al., 2019). However, TT8 orthologs in different species may also perform different functions. Jia et al. (2021) found that *DcTT8* from *Dendrobium candidum* could directly activate the expression of *DcF3′H* and *DcUFGT* by binding to their promoters. Zhai et al. (2020) found that targeted mutations of *BnTT8* blocked PA-specific deposition, but elevated seed oil content and altered fatty acid (FA) composition in the seed coat of *Brassica napus*. In addition, bHLHs can also negatively regulate anthocyanin biosynthesis. To data, some *bHLH* genes have been reported as repressors of anthocyanin biosynthesis in plants, including IN1 from maize (Burr et al., 1996), *bHLH3*/*13*/14/17 from *Arabidopsis* (Song et al., 2013), *LcbHLH92* from *Leymus chinensis* (Zhao et al., 2018), and *CpbHLH1* from *Chimonanthus praecox* (Zhao et al., 2020), but the molecular mechanisms of their negative regulation have been little reported.

Eggplants (*Solanum melongena*) with purple peel are rich in anthocyanins and popular with consumers. They rank fourth in facility vegetable cultivation planting area. Noda et al. (2000) reported that eggplant extracts showed the most potent superoxide anion radical scavenging activity (SOD-like activity) of 16 common vegetables examined, highlighting the important role of anthocyanins in eggplant. Recently, research focused on the molecular mechanisms of anthocyanin biosynthesis in eggplant has increased (Jiang et al., 2016; Li et al., 2017, 2018; Moglia et al., 2020; Zhou et al., 2020), but gene functions have been verified primarily by ectopic expression in *Arabidopsis* or tobacco. The eggplant genome database was updated by Barchi et al. (2019), providing a valuable resource for the molecular breeding of new eggplant germplasm. Based on the updated eggplant genome, Moglia et al. (2020) functionally characterized the genes belonging to the MBW complex (*SmelANT1*, *SmelAN2*, *SmelJAF13*, and *SmelAN1*), and identified out an R3 MYB type repressor (*SmelMYBL1*) of anthocyanin biosynthesis.

Based on the two published eggplant genome databases (Hirakawa et al., 2014; Barchi et al., 2019), *SmbHLH1* (*Sme2.5_00592.1_g00005.1*) and *SmTT8* (*SMEL_009g326640.1*) were identified as two TT8 homologs. SmTT8 has been shown to interact with SmMYB75 to promote anthocyanin biosynthesis in eggplant (Shi et al., 2021). However, no studies on *SmbHLH1* have been reported. Hence, we overexpressed *SmbHLH1* in eggplant, and obtained two stable transformation lines, *SmbHLH1*-1 and *SmbHLH1*-2. The anthocyanin content was markedly reduced in both lines, indicating that *SmbHLH1* and *SmTT8* have different functions and molecular mechanisms in the regulation of anthocyanin biosynthesis in eggplant. We used biochemical experiments and transcriptomic analysis to investigate the molecular mechanism by which *SmbHLH1* regulates anthocyanin biosynthesis.

## Materials and Methods

### Plant Materials and Growth Conditions

Six eggplant cultivars with different color and anthocyanin contents in the fruit and stem peels, named No. 44, No. 64, No. 76, No. 108, No. 109, and No. 133 (Supplementary Figure S1), were grown in the solar greenhouse of Shandong Agricultural University to perform phenotypic observations and collect experimental materials. No. 109 was used for genetic transformation.

*Nicotiana benthamiana* was grown in a phytotron under 25/16°C and 12/12h day/night conditions. Plants with 5–6 leaves were used for bimolecular fluorescence complementation (BiFC), dual luciferase, and transient expression assays.

### Anthocyanins Content Analysis

The anthocyanin content was measured with the methods described by Neff and Chory (1998).

### Gene Isolation and Sequence Analysis

The full-length coding sequences (CDSs) of *SmbHLH1* and *SmTT8* were cloned by PCR amplification. The amino acid sequences of SmbHLH1 and SmTT8 were translated with DNAMAN 6.0 (Lynnon Biosoft, Quebec, Canada) based on the sequencing results. Multiple sequence alignments were performed using ClustalX (version 1.83) and DNAMAN 6.0. The MEGA 7.0 program was used to construct neighbor-joining phylogenetic trees with the following parameters: bootstrapping (1,000 replicates, random seed), Poisson model, and complete deletion (Kumar et al., 2016).

### Subcellular Localization

The full-length CDS of *SmbHLH1* without the stop codon was inserted into the pHB:GFP vector. The fusion construct was transferred into *Agrobacterium tumefaciens* strain GV3101, and subcellular localization assays were performed as previously reported (Sparkes et al., 2006).

### Yeast Two-Hybrid Assay

The full-length CDSs of *SmbHLH1* and *SmTT8* were inserted into the pGBKT7 vector as bait, and *SmMYB113* was fused to the pGADT7 vector as prey. The Y2H assays were performed as described in Zhou et al. (2020).

### Bimolecular Fluorescence Complementation

The full-length CDSs of *SmbHLH1* and *SmTT8* without stop codons were inserted into the pXY104 vector, and *SmMYB113* with the stop codon was subcloned into the pXY106 vector. BiFC assays were performed as described by Li et al. (2016a).

### Plasmid Construction and Plant Transformation

The full-length CDS of *SmbHLH1* was inserted into the pRI 101 vector with the 35S-CaMV promoter. The fusion vector was transferred into *Agrobacterium* strain LBA4404 and introduced into No. 109 by *Agrobacterium*-mediated cotyledon explant transformation based on an earlier report by Vasudevan et al. (2005) with minor modifications.

### Dual Luciferase and Transient Expression Assays in *N. benthamiana* Leaves

The complete coding regions of *SmMYB113*, *SmbHLH1*, and *SmTT8* were inserted into the pHB vector, and the promoters of anthocyanin biosynthetic genes (*SmCHS*, *SmCHI*, *SmF3H*, *SmF3′H*, *SmF3′5′H*, *SmDFR*, *SmANS*, and *Sm3GT*) were amplified and cloned into pGreenII 0800-LUC vectors. The Dual-LUC assay was performed as previously reported (Hu et al., 2019). For the transient expression assays, 1/2×, 1×, and 2× pHB-*SmbHLH1* was co-infiltrated with pHB-*SmMYB113* or pHB-*SmMYB113*+pHB-*SmTT8*. The anthocyanin content was measured 4days after infiltration with the methods described in Li et al. (2017).

### Quantitative Reverse Transcription PCR

The fruit and stem peels of six eggplant cultivars and thetender stems close to the growing point of No.109 were collected, and total RNA was extracted from the samples using the TaKaRa MiniBEST Plant RNA Extraction Kit (TaKaRa, Otsu, Shiga, Japan) according to the manufacturer’s instructions. Then 1μg RNA was used for cDNA synthesis with the PrimeScript RT Reagent Kit with gDNA Eraser (TaKaRa). The quantitative rreverse ttranscription PCR (RT-qPCR) assay was performed according to the manufacturer’s instructions of the SYBR Premix Ex Taq II Kit (TaKaRa) on the LightCycler 96 system (Roche, Basel, Switzerland). The *Actin* gene (GU984779.1) was used as an internal control gene. The relative expression levels of the amplified products were analyzed using the comparative CT method (Livak and Schmittgen, 2001).

### RNA-Seq and Bioinformatics Analyses

Tender stems close to the growing point were collected from WT and two *SmbHLH1* transgenic eggplants for RNA-seq. RNA extraction, quality control, library construction, and high-throughput parallel sequencing were performed at the Beijing Genomics Institute (BGI) on the BGISEQ-500 sequencing platform. Bioinformatics analyses were performed as previously reported (Li et al., 2019).

### Statistical Analysis

SPSS 17.0 (SPSS, Inc., Chicago, United States) was used to assess statistical significance by one-way ANOVA and Duncan’s New Multiple Range test (*p*≤0.05).

## Results

### Molecular Cloning and Characterization of TT8 Homologs in Eggplant

**Figure 1:**
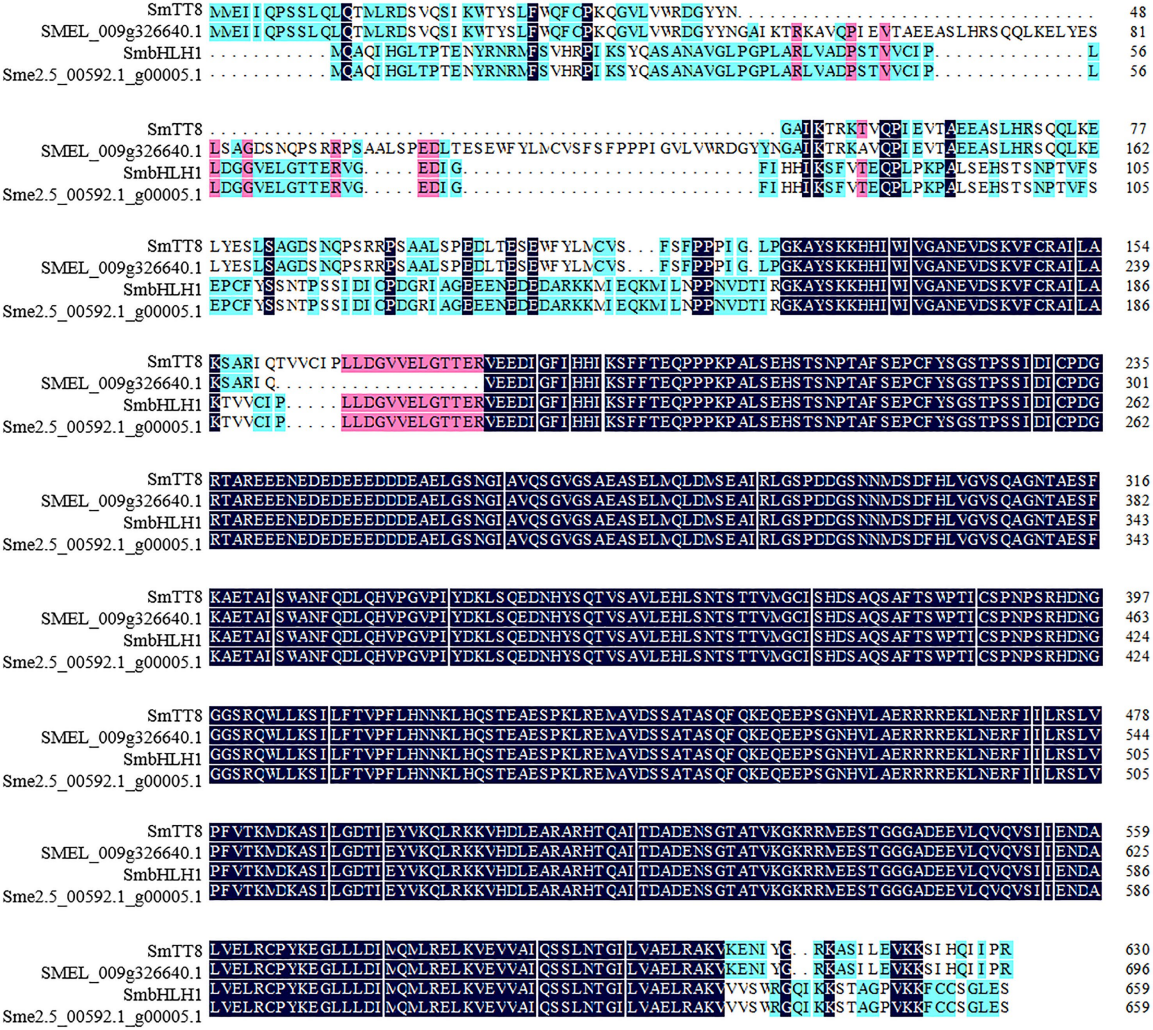
Multiple sequence alignments of the amino acids of SmbHLH1 and SmTT8. SMEL_009g326640.1 and Sme2.5_00592.1_g00005.1 were both annotated as TT8 in the two published eggplant genome databases.

The CDSs of *SmbHLH1* and *SmTT8* were amplified based on the two published eggplant genome databases (Hirakawa et al., 2014; Barchi et al., 2019). After sequencing and alignment, we found that the *SmTT8* sequence showed some differences from the published sequence (*SMEL_009g326640.1*), whereas *SmbHLH1* was identical to *Sme2.5_00592.1_g00005.1* (Supplementary Figure S2). We then aligned the amino acid sequences of SmbHLH1 and SmTT8 (Figure 1) and found that only the regions at the N terminus (amino acids 1–158 and 188–193) and C terminus (amino acids 633–659) differed between SmbHLH1 and SmTT8.

A phylogenetic tree was constructed to analyze the relationships of SmbHLH1 and SmTT8 with all bHLH proteins from *Arabidopsis*, *Solanum lycopersicum*, *Malus* × *domestica*, *Vitis vinifera*, *Fragaria* × *ananassa*, *Petunia* × *hybrida*, *Prunus persica*, *Chimonanthus praecox*, and *Medicago truncatula* known to be involved in the anthocyanin biosynthesis pathway (Supplementary Figure S3; Li et al., 2017; Zhao et al., 2020). As shown in Supplementary Figure S3, SmbHLH1 and SmTT8 were placed in the same clade.

### Different Expression Pattern of *SmbHLH1* and *SmTT8* in Eggplant

**Figure 2:**
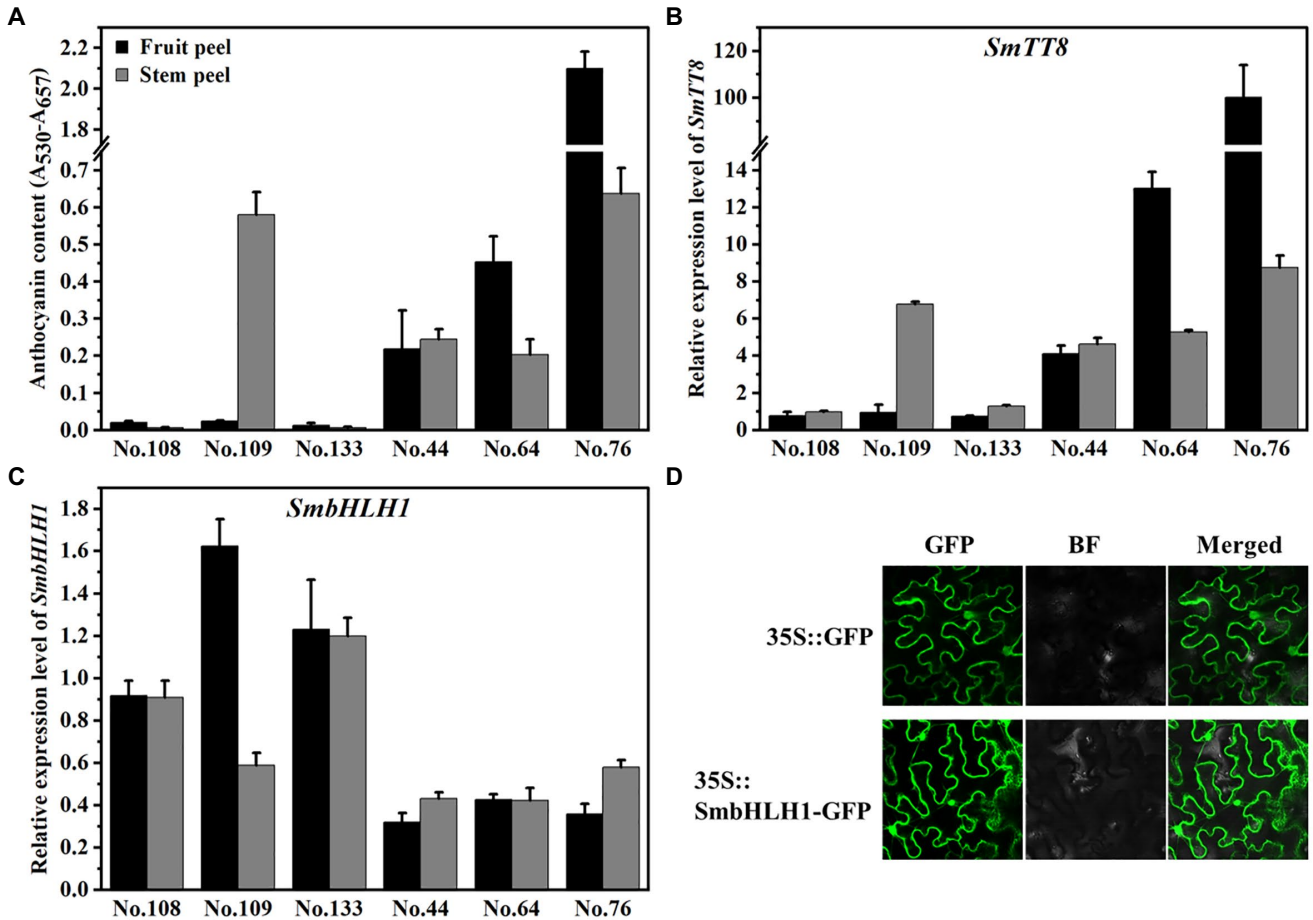
Relationship of anthocyanin contents with the expression of *SmTT8* and *SmbHLH1*. **(A)** The anthocyanin contents in the fruit and stem peels of six eggplant cultivars; **(B,C)** The expression levels of *SmTT8* and *SmbHLH1* in the fruit and stem peels of six eggplant cultivars. Bars represent means±SD of three biological replicates; and **(D)** Subcellular localization analysis of SmbHLH1.

To analyze the relationship between *SmbHLH1* and *SmTT8*, the expression pattern of *SmbHLH1* and *SmTT8* were measured in the fruit and stem peels from six eggplant cultivars with different anthocyanin contents (Figure 2A; Supplementary Figure S1). The primers for RT-qPCR were designed from the 5′ termini, which differed between *SmbHLH1* and *SmTT8* (Supplementary Table S1). As shown in Figure 2A and Supplementary Figure S1, the anthocyanin content in the fruit peel was sorted as No. 76>No. 64>No. 44>No. 108/No. 109/No. 133, while No. 76/No. 109>No. 44>No. 64>No. 108/No. 133 in the stem peel. The expression patterns of *SmTT8* in the fruit and stem peel were both consistent with anthocyanin contents (Figure 2B). However, the expression pattern of *SmbHLH1* was opposite with anthocyanin contents in both fruit and stem peel (Figure 2C). These results implied that the function of *SmbHLH1* may be different from that of *SmTT8*.

To confirm the subcellular location of *SmbHLH1*, the 35S::SmbHLH1-GFP fusion protein was constructed and transiently expressed in tobacco leaves. Compared with the positive control, SmbHLH1-GFP was predominately present in the cytosol and nucleus (Figure 2D).

### Overexpression of *SmbHLH1* Inhibited Anthocyanin Accumulation in Eggplant

**Figure 3:**
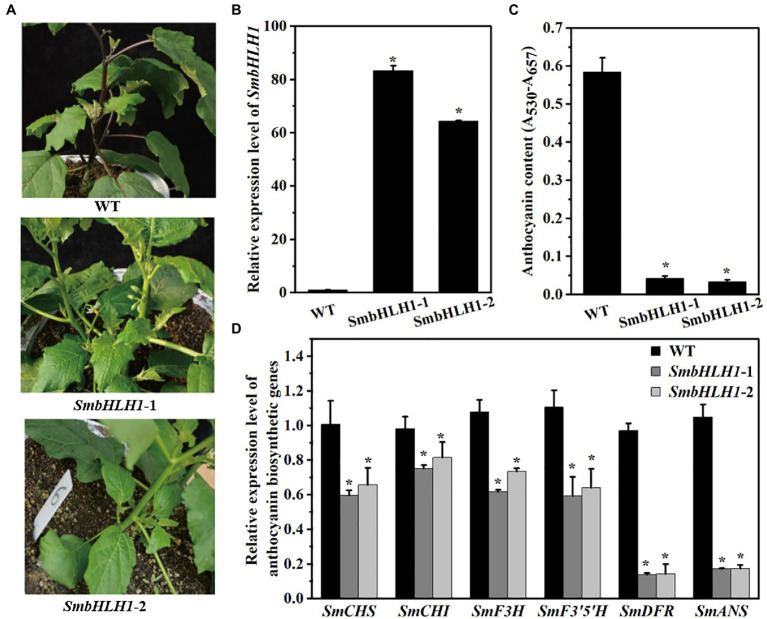
*SmbHLH1* controls anthocyanin biosynthesis in the stem peels of eggplant (cultivar No. 109). **(A)** The phenotypes of WT and the two *SmbHLH1* transgenic lines (*SmbHLH*-1 and -2); **(B)** The relative expression level of *SmbHLH1* in the two 35S::*SmbHLH1* stem peels lines; **(C)** The anthocyanin content in the two 35S::*SmbHLH1* stem peels lines; and **(D)** The relative expression level of anthocyanin biosynthetic genes in stem peels of the two 35S::*SmbHLH1* lines. Values are means±SD (*n*=3). * represents significance at *p*<0.05 comparing with wild-type (WT).

To characterize the function of *SmbHLH1* in eggplant, transient VIGS assay, CRISPR-Cas9 system, and overexpression system were all performed on all the six eggplant cultivars. However, the approaches to transform the eggplant with the transient VIGS assay and CRISPR-Cas9 system were failed. So we decided to transform the eggplant with 35S promoter-constructs to probe the overall function of the *SmbHLH1* gene. *SmbHLH1* was introduced into eggplant by *Agrobacterium*-mediated transformation. Only the eggplant cultivar No.109 differentiated two *SmbHLH1* overexpression transgenic lines (overexpressed by the 35S-CaMV promoter, named as *SmbHLH1*-1 and *SmbHLH1*-2), which would be used to probe the overall function of the *SmbHLH1*. Both of the two 35S::*SmbHLH1* transgenic eggplant lines showed increased *SmbHLH1* expression (Figures 3A,B). As shown in Figure 3A, the whole plant of the two 35S::*SmbHLH1* transgenic eggplant lines were greener than WT. Especially, the colors of stalks, sepals, and stem peels were changed from purple to green, which could be accumulated anthocyanins in cultivar No.109. However, the fruit peels of cultivar No.109 was orange with little anthocyanins, no significant difference was found comparing with the two *35S*::*SmbHLH1* transgenic eggplant lines. Next, we evaluated the anthocyanin contents and the expression levels of anthocyanin biosynthetic genes (*SmCHS*, *SmCHI*, *SmF3H*, *SmF3′5′H*, *SmDFR*, and *SmANS*) in the stems peel. Anthocyanin contents and expression levels of anthocyanin biosynthetic genes were lower in stems peel of the two *SmbHLH1* transgenic lines than in WT stems (Figures 3C,D).

### SmbHLH1 Could Not Interact With SmMYB113

**Figure 4:**
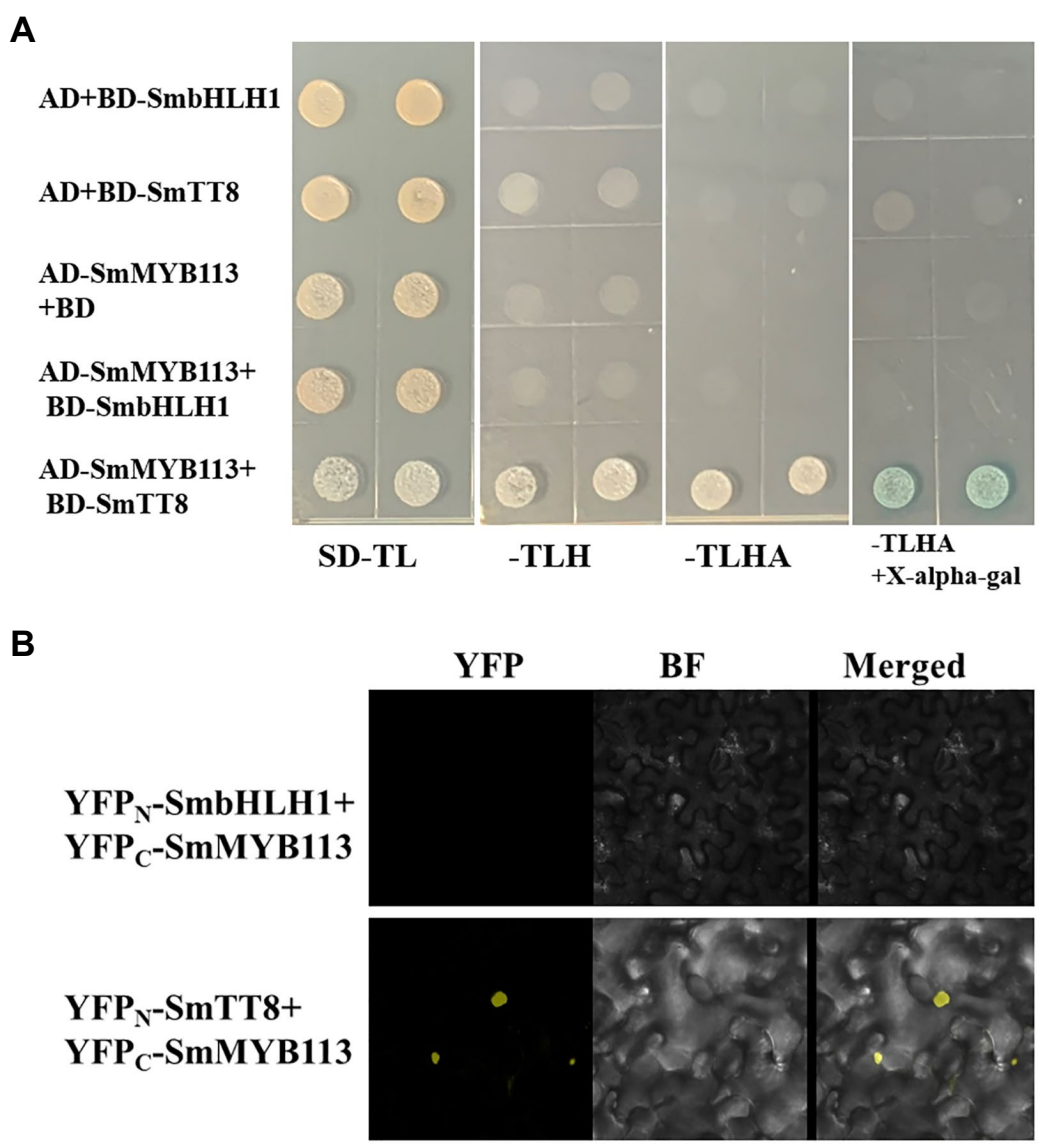
SmbHLH1 could not specifically interact with SmMYB113 in both Y2H and bimolecular fluorescence complementation (BiFC) assays, while SmTT8 could. **(A)** The yeast cells of strain AH109 harboring the indicated plasmid combinations were grown in SD-TL (−Trp/−Leu), SD-TLH (−Trp/−Leu/-His), SD-TLHA (−Trp/−Leu/-His/−Ade) mediums, and SD-TLHA (−Trp/−Leu/-His/−Ade) with X-alpha-gal filter assay were indicated. **(B)** YFP fluorescent and bright-field images of *Nicotiana benthamiana* leaf cells infiltrated with a mixture of *Agrobacterium* suspensions harboring constructs encoding the indicated fusion proteins.

We performed yeast two-hybrid (Y2H) assays to determine whether SmbHLH1 regulated anthocyanin biosynthesis through interaction with SmMYB113, which has been reported as a critical anthocyanin regulator and interacted with SmTT8 (Zhou et al., 2020). Positive β-gal activity was observed only in yeast that contained AD-SmMYB113 plus BD-SmTT8 grown on -T/−L/-H/−A screening medium but not in yeast that contained AD plus BD-SmbHLH1, AD plus BD-SmTT8, AD-SmMYB113 plus BD, or AD-SmMYB113 plus BD-SmbHLH1 (Figure 4A). These results indicated that SmbHLH1 could not interact with SmMYB113, whereas SmTT8 could.

Bimolecular fluorescence complementation assays were performed to further characterize the interaction between SmbHLH1/SmTT8 and SmMYB113 *in vivo*. As shown in Figure 4B, YFP fluorescence signals appeared only when YFP_C_-SmMYB113 and YFP_N_-SmTT8 were co-expressed. No fluorescence was detected in cells that contained combinations of YFP_C_-SmMYB113 plus YFP_N_-SmbHLH1 and other empty vector controls. In addition, we found that the interaction location of SmMYB113 and SmTT8 complexes was in the nucleus. These results confirmed that SmbHLH1 could not interact with SmMYB113 in plant cells, but SmTT8 could.

### SmbHLH1 Is a Negative Transcription Regulator of *SmDFR* and *SmANS*

**Figure 5:**
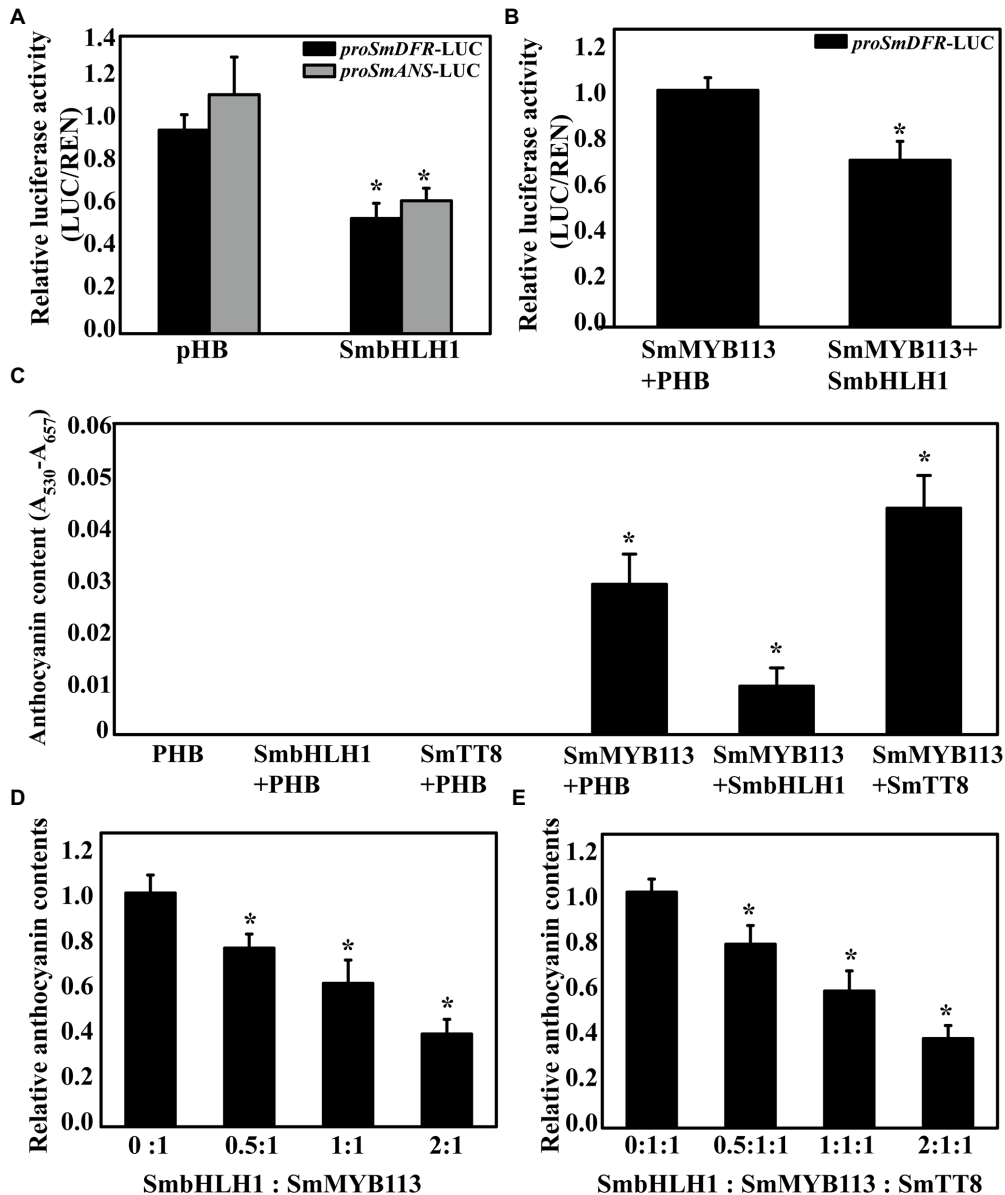
Dual-luciferase and transient analysis of SmbHLH1 on anthocyanin accumulation. **(A)** Inhibition of the activity of the *SmDFR* and *SmANS* gene promoters by SmbHLH1 in transient expression assay in *N. benthamiana* leaves; **(B)** SmbHLH1 decreased the ratio of LUC/REN of the *SmDFR* promoter activated by SmMYB113; and **(C)** SmbHLH1 inhibited SmMYB113-induced anthocyanin accumulation while SmTT8 enhanced in *N. benthamiana* leaves. **(D,E)** The relative anthocyanin contents induced by different proportions of *SmbHLH1*:*SmMYB113* and *SmbHLH1*:*SmMYB113*:*SmTT8* in infiltrated regions. *Represents significance at *p* < 0.05 comparing with the control.

The *cis*-elements in the promoter sequences of six anthocyanin biosynthetic genes (*SmCHS*, *SmCHI*, *SmF3H*, *SmF3′5′H*, *SmDFR*, and *SmANS*) were analyzed, and E-box or G-box *cis*-elements recognized by bHLH family members were found in all six promoter sequences (Supplementary Figure S4). Then, we performed a dual-luciferase assay by infiltration of tobacco leaves to analyze whether SmbHLH1 can inhibit the transcription of the anthocyanin biosynthetic genes. As a result, we found that *SmDFR* and *SmANS* promoter activities were inhibited by SmbHLH1 (Figure 5A). Because SmMYB113 can bind directly to the *SmDFR* promoter and activate its expression (Jiang et al., 2016), *SmMYB113* was co-infiltrated with *SmbHLH1*. As shown in Figure 5B, the inhibition of the *SmDFR* promoter activity by SmbHLH1 occurred in the presence of SmMYB113.

Another transient expression assay was carried out in tobacco leaves to explore the role of *SmbHLH1* in anthocyanin biosynthesis. *Agrobacterium tumefaciens* GV3101 strains containing the empty vector pHB, *SmbHLH1*, *SmTT8*, or *SmMYB113* were infiltrated or co-infiltrated into tobacco leaves. As shown in Figure 5C, pigmentation was evident after infiltration with *SmMYB113* alone and co-infiltration with *SmbHLH1* and *SmTT8*. Compared with the leaves infiltrated with *pHB* and *SmMYB113* alone, anthocyanin content decreased when SmbHLH1 was added but increased when SmTT8 was added. Next, different ratios of *SmbHLH1*:*SmMYB113* and *SmbHLH1*:*SmMYB113*:*SmTT8* were used to analyze the effect of SmbHLH1 on SmMYB113-induced anthocyanin biosynthesis (Figures 5D,E). We found that anthocyanin accumulation in infiltrated patches was negatively correlated with the dose of *SmbHLH1*. These results suggested that SmbHLH1 was a key negative regulator of anthocyanin accumulation in eggplant, directly inhibiting *SmDFR* and *SmANS* expression through a SmMYB113-independent pathway.

### Overview of RNA-Seq Analysis

**Figure 6:**
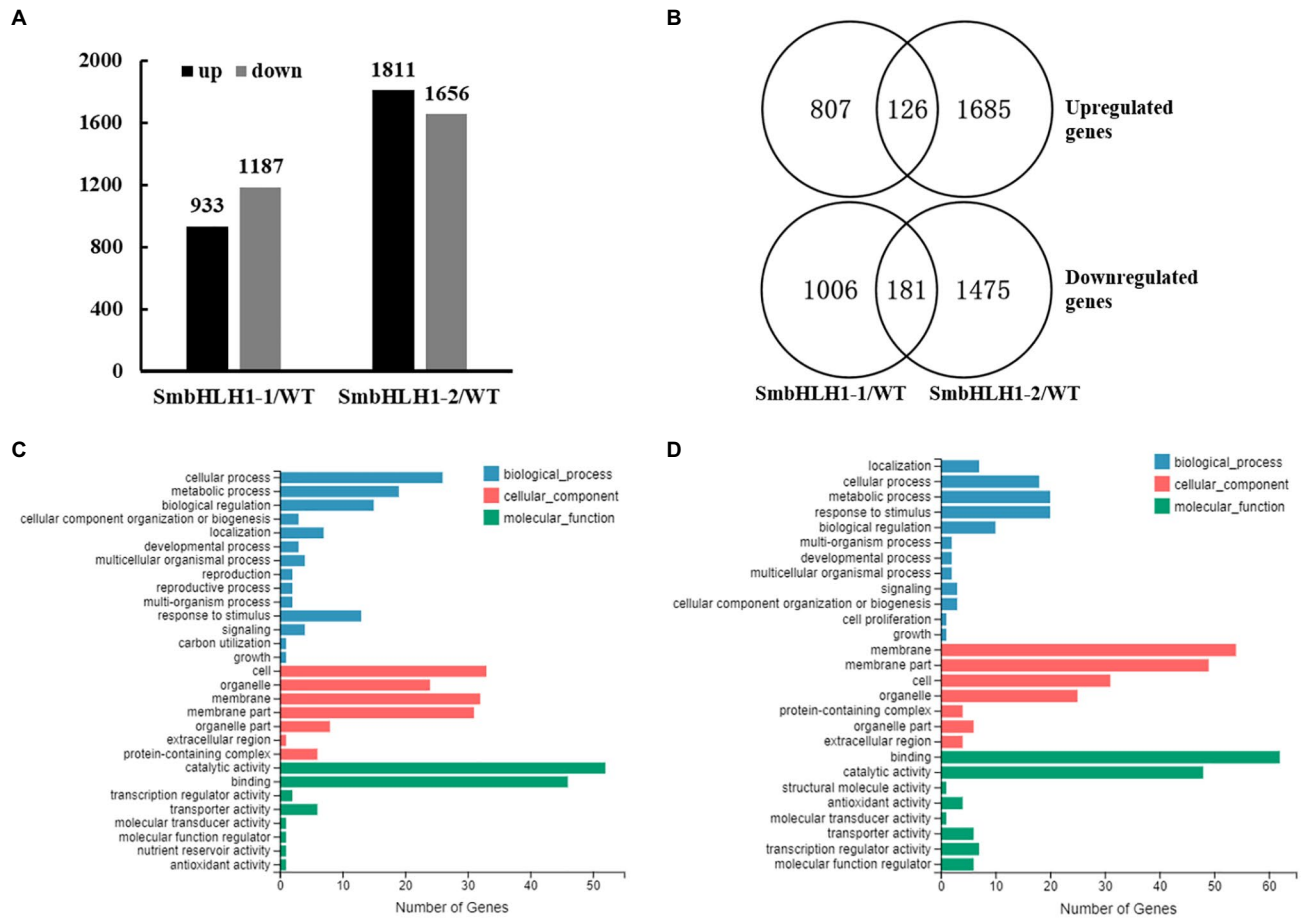
Overview, Venn diagrams and GO enrichment analysis of up- or downregulated genes by SmbHLH1 in stems at a level |log_2_^ratio^|≥0.5 with *p*<0.001. **(A)** Number of upregulated and downregulated genes in the two *35S::SmbHLH1* stem peels lines vs. WT with RNA-seq. **(B)** Schematic of the Venn diagram analysis of common upregulated and downregulated genes [differentially expressed genes (DEGs)] in the two *35S::SmbHLH1* stem peels lines vs. WT. **(C,D)** GO functional enrichment analysis of common upregulated **(C)** and downregulated **(D)** genes.

To explore how SmbHLH1 regulates gene expression at the whole-genome level, total RNA was extracted from stems of the WT and two *SmbHLH1* transgenic lines for RNA-seq analysis. A total of 29,840 genes or transcripts were obtained. Subsequently, we performed pairwise comparisons of transcript abundance to identify differentially expressed genes (DEGs) between the WT and transgenic lines based on an absolute fold change value of |log_2_^ratio^|≥0.5 with *p*<0.001. In total, 2,120 genes were differentially expressed in *SmbHLH1*-1 stems compared with the WT (933 up; 1,187 down), and 3,467 genes were differentially expressed in *SmbHLH1*-2 stems compared with the WT (1,811 up; 1,656 down; Figure 6A; Supplementary Table S2). Among these DEGs, only 126 genes were upregulated and 181 genes were downregulated simultaneously in both *SmbHLH1*-overexpressing lines compared with the WT (Figure 6B).

GO functional enrichment analysis was performed on the 126 upregulated and 181 downregulated genes shared by the two SmbHLH1 transgenic eggplant lines (Figures 6C,D). The 126 upregulated genes were enriched in 29 GO terms, and the 181 downregulated genes were enriched in 27 GO terms. In the “biological process” category, “cell proliferation” was enriched only in the downregulated genes, and “carbon utilization,” “reproduction,” and “reproductive process” were enriched only in the upregulated genes. In the “molecular function” category, “nutrient reservoir activity” was enriched only in the upregulated genes, and “structural molecule activity” was enriched only in the downregulated genes.

The Kyoto Encyclopedia of Genes and Genomes (KEGG) pathways of the 126 shared upregulated genes and the 181 shared downregulated genes were identified using a value of *p* less than 0.05 as the cut-off (Table 1). As shown in Table 1, the “carbon metabolism” and “starch and sucrose metabolism” pathways were enriched in the upregulated genes, and the “flavonoid biosynthesis” and “anthocyanin biosynthesis” pathways were enriched in the downregulated genes. These results suggest that SmbHLH1 inhibited anthocyanin biosynthesis but promoted carbon metabolism. We next examined glucose, fructose, and sucrose contents in stems, functional leaves, and expanding leaves. The two *SmbHLH1* transgenic eggplant lines showed significant increases in glucose, fructose, and sucrose content compared with the WT (Supplementary Figure S5). These results are consistent with the transcriptomic analysis.

**Table 1:** Kyoto Encyclopedia of Genes and Genomes (KEGG) pathway enrichment analysis of common upregulated and downregulated genes in two SmbHLH1 transgenic eggplant stem peels lines vs. WT.

Upregulated genes	Downregulated genes
ko01100 Metabolic pathways (26) ko01110 Biosynthesis of secondary metabolites (15) ko01200 Carbon metabolism (7) ko:K00382 DLD; dihydrolipoamide dehydrogenase ko:K00873 PK; pyruvate kinase ko:K01602 rbcS; ribulose-bisphosphate carboxylase small chain ko:K01913 AAE7; acetate/butyrate---CoA ligase ko:K02437 gcvH; glycine cleavage system H protein ko:K03841 FBP; fructose-1,6-bisphosphatase I ko:K05605 HIBCH; 3-hydroxyisobutyryl-CoA hydrolase ko04075 Plant hormone signal transduction (5) ko04016 MAPK signaling pathway – plant (5) ko04626 Plant-pathogen interaction (4) ko00630 Glyoxylate and dicarboxylate metabolism (4) ko00010 Glycolysis/Gluconeogenesis (4) ko00500 Starch and sucrose metabolism (2) ko:K01177 E3.2.1.2; beta-amylase ko:K01179 E3.2.1.4; endoglucanase	ko01100 Metabolic pathways (31) ko01110 Biosynthesis of secondary metabolites (19) ko00564 Glycerophospholipid metabolism (6) ko04141 Protein processing in endoplasmic reticulum (5) ko00941 Flavonoid biosynthesis (5) ko:K00475 F3H; Naringenin 3-dioxygenase ko:K00660 CHS; Chalcone synthase (CHS) ko:K05277 ANS; Anthocyanidin synthase ko:K13065 E2.3.1.133; Shikimate O-hydroxycinnamoyl transferase ko:K13082 DFR; bifunctional dihydroflavonol 4-reductase (DFR)/flavanone 4-reductase ko04075 Plant hormone signal transduction (4) ko00942 Anthocyanin biosynthesis (1) ko:K12930 BZ1; Aanthocyanidin 3-O-glucosyltransferase

## Discussion

The bHLH, R2R3-MYB, and WD repeat proteins can form a ternary MBW complex to modulate the expression of anthocyanin biosynthesis-related genes. Previous studies have revealed that several bHLH TFs, including AtTT8, AtGL3, AtEGL3, PhAN1, PhJAF13, GhMYC1, SlAN1, MdbHLH3, and PbbHLH2, interact with their respective MYB partners AtPAP1, AtTT2, PhAN2, GhMYB10, SlAN2, MdMYB1, and PbMYB9/10/10b to regulate anthocyanin or proanthocyanidin biosynthesis in *Arabidopsis*, *Petunia hybrida*, *Gerbera hybrida*, *S. lycopersicum*, *M. domestica*, and *Pyrus bretschneideri* (Elomaa et al., 2003; Koes et al., 2005; Ramsay and Glover, 2005; Xie et al., 2012; Sun et al., 2020a; Li et al., 2021b). Xie et al. (2012) reported that two regions (amino acids 1–24 and 186–228) at the *N* terminus of MdbHLH3 were crucial for the interaction between MdbHLH3 and MdMYB1. In this study, we found that SmbHLH1, SmTT8, and MdbHLH3 were clustered into one clade by phylogenetic analysis (Supplementary Figure S3), and the *N* terminus (amino acids 1–158 and 188–193) and C terminus (amino acids 633–659) of SmbHLH1 differed significantly from those of SmTT8 (Figure 1). SmbHLH1 did not interact with SmMYB113 in both Y2H and BiFC assays (Figure 4), whereas SmTT8 did which was corresponding to the results reported by Moglia et al. (2020). These results suggested that the N-terminal portion of SmTT8 was crucial for its interaction with SmMYB113. Simultaneously, the pattern of *SmbHLH1* expression contrasted with those of *SmTT8* expression and anthocyanin accumulation (Figure 2). Previous studies have reported that the enhancement of anthocyanin biosynthesis by SmTT8 is dependent on SmMYB113 (Moglia et al., 2020; Zhou et al., 2020). However, we found that over-expression of *SmbHLH1* inhibited anthocyanin accumulation (Figure 3). Dual-luciferase assays revealed that SmbHLH1 directly repressed the expression of *SmDFR* and *SmANS* (Figure 5). Because the color of cultivar No.109 tissues was different, the phenotype changes of *35S*::*SmbHLH1* transgenic eggplant lines were different. For the tissues with anthocyanins accumulation were changed from purple to green, such as stalks, sepals, and stem peels. However, no significant color change was found in the tissues with little anthocyanins content, such as the fruit peels. Taken all above, we could conclude that *SmbHLH1* was a repressor in anthocyanin biosynthesis. Moreover, the silencing of *SmbHLH1* in fruit peel tissue to verify the fruit can accumulate more anthocyanins is worthy in the future,

BHLH proteins are the second largest class of transcription factors in plants and can be divided into 26–32 subgroups (Carretero-Paulet et al., 2010; Pires and Dolan, 2010); they play important roles in stress tolerance, reproduction, and secondary metabolite biosynthesis (Sun et al., 2020b; Guo et al., 2021). It is worth noting that AtTT8, AtGL3, AtEGL1, and AtMYC1, which are positive regulator of anthocyanin biosynthesis, were placed into subgroup IIIf (Heim et al., 2003). However, *AtMYC1* orthologs in maize and *C. praecox*, as well as another bHLH TF from subgroup IVd, have been reported to negatively regulate anthocyanin biosynthesis (Burr et al., 1996; Zhao et al., 2018). Here, *SmTT8* and *SmbHLH1* are both eggplant orthologs of *AtTT8*, but they played opposite roles in anthocyanin biosynthesis. It is not uncommon for such homologous genes to exhibit different functions in the plant. For example, Li et al. (2021a) reported that the biological function of MS1 in pollen development differed in the evolution of Poaceae plants. The different biological functions of homologous genes probably result from differences in some amino acids. Taking all of our results into consideration, we speculate that the N-terminal front portion of SmbHLH1 may be the key to its role in anthocyanin biosynthesis.

RNA-seq analysis revealed that overexpression of *SmbHLH1* reduced the expression of anthocyanin biosynthetic genes while enhancing that of genes involved in carbon metabolism and starch and sucrose metabolism. Combined with another phenotype of the two *SmbHLH1* transgenic eggplant lines, which were greener than the WT due to increased chlorophyll content (Figure 3; Supplementary Figure S4), we speculated that more light was absorbed, resulting in significant increases in glucose, fructose, and sucrose content. Anthocyanin and chlorophyll are two major plant pigments. Anthocyanin can protect plants from UV light and oxidative damage, whereas chlorophyll is essential for the conversion of light energy into stored chemical energy. Previous studies have reported that anthocyanin often accumulates in young leaves but degrades in mature leaves, which is beneficial for the gradual accumulation of chlorophyll in many plants (Oren-Shamir, 2009; Ren et al., 2019). On this basis, we speculate that the increased chlorophyll content may be directly regulated by *SmbHLH1* or caused by a decrease in anthocyanin, suggesting the existence of a much more complex molecular mechanism that is worthy of study in the future.

## Conclusion

In the eggplant genome, we found that both SmbHLH1 and SmTT8 are homologs of TT8. Their amino acid sequences differed only at the N and C termini, but they had contrasting functions in anthocyanin biosynthesis. SmbHLH1 repressed the expression of *SmDFR* and *SmANS* and inhibited anthocyanin biosynthesis through SmMYB113-independent pathway. We identified *SmbHLH1* as a new target to silence to produce more anthocyanins in eggplant fruit peel. In addition, RNA-seq analysis revealed that genes involved in carbon metabolism and starch and sucrose metabolism were upregulated by overexpression of *SmbHLH1* in eggplant.

## Data Availability Statement

The datasets presented in this study can be found in online repositories. The names of the repository/repositories and accession number(s) can be found at: NCBI BioProject, PRJNA761256.

## Author Contributions

FY, JL, and MW designed the research. ZD, ST, JL, and GY performed experiments. JL and ZD analyzed data. JL wrote the manuscript. FY and JL read and modified the manuscript. All authors contributed to the article and approved the submitted version.

## Funding

This work was supported by the National Natural Science Foundation of China (grant number: 31672169), Youth Project of Shandong Provincial Natural Science Foundation (grant number: ZR201911120670), Key Project of Shandong Provincial Natural Science Foundation (ZR2020KC039), and Special Project Protected Horticulture Advantage Team of Shandong Agricultural University ‘Double First-Class’ Science and Technology Team (grant number: SYL2017YSTD07).

## Conflict of Interest

The authors declare that the research was conducted in the absence of any commercial or financial relationships that could be construed as a potential conflict of interest.

## Publisher’s Note

All claims expressed in this article are solely those of the authors and do not necessarily represent those of their affiliated organizations, or those of the publisher, the editors and the reviewers. Any product that may be evaluated in this article, or claim that may be made by its manufacturer, is not guaranteed or endorsed by the publisher.
