# Supplementary material for: The Basic Helix-Loop-Helix Transcription Factor SmbHLH1 Represses Anthocyanin Biosynthesis in Eggplant
Source: Front Plant Sci. 2021 Nov 12;12:757936. doi: 10.3389/fpls.2021.757936 (PMC8633956; doi:10.3389/fpls.2021.757936)
Supplement: Supplementary file 1 [file Data_Sheet_1.docx]

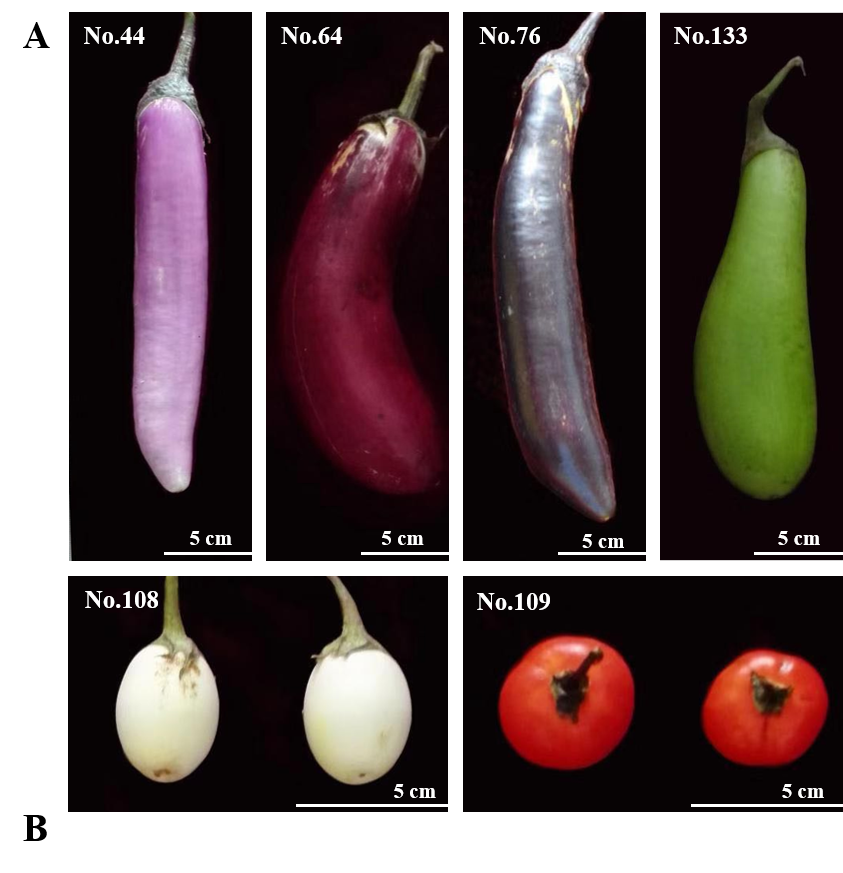


**Supplementary Figure 1.** The fruit photos of six eggplant cultivars.

**Supplementary Figure 2.** Multiple sequence alignments of the coding sequences of *SmTT8* and *SmbHLH1*.


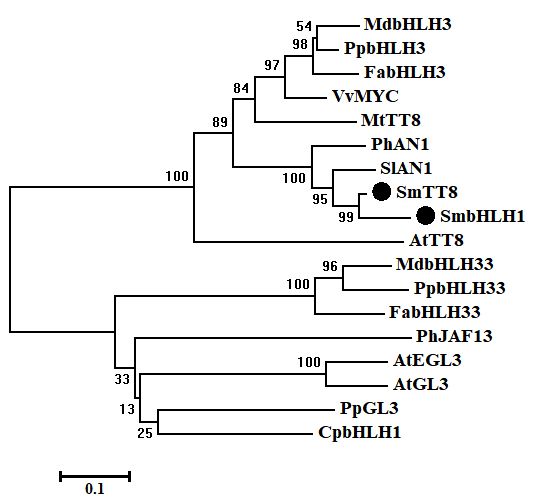


**Supplementary Figure 3.** Phylogenetic relationships of the SmbHLH1 and SmTT8 with bHLHs from other species known to be involved in the flavonoid biosynthesis pathway. Protein sequences are from GenBank with the following accession numbers: *Malus* × *domestica* MdbHLH3 (ADL36597), MdbHLH33 (ABB84474); *Prunus persica* PpbHLH3 (AIE57508), PpbHLH33 (XP_007220209), PpGL3 (XP_020420271); *Fragaria* × *ananassa* FabHLH3 (AFL02463), FabHLH33 (AFL02465); *Vitis vinifera* VvMYC (NP_001268182); *Medicago truncatula* MtTT8 (KM892777); *Petunia* × *hybrid* PhAN1 (AAG25927.1), PhJAF13 (AAC39455); *Solanum lycopersicum* SlAN1 (ALC74034); *Arabidopsis* AtTT8 (Q9FT81), AtEGL3 (Q9CAD0), AtGL3 (Q9FN69); *Chimonanthus praecox* CpbHLH1 (QKL20124). Protein sequences were analyzed using MEGA7.0 and the neighbor‐joining method with 1000 bootstrap replicates.


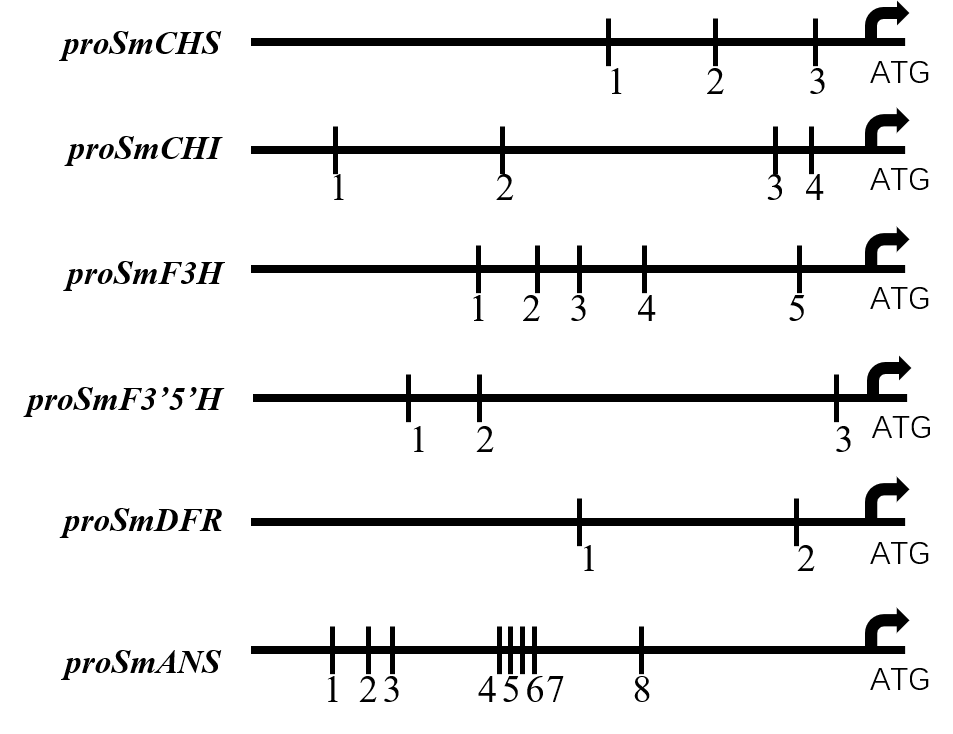


**Supplementary Figure 4.** Distribution of E-box (5′-CANNTG-3′) or G-box(5′-CACGTG-3′) cis-elements on the promoters (2000bp upstream of ATG) of the anthocyanin biosynthetic genes.


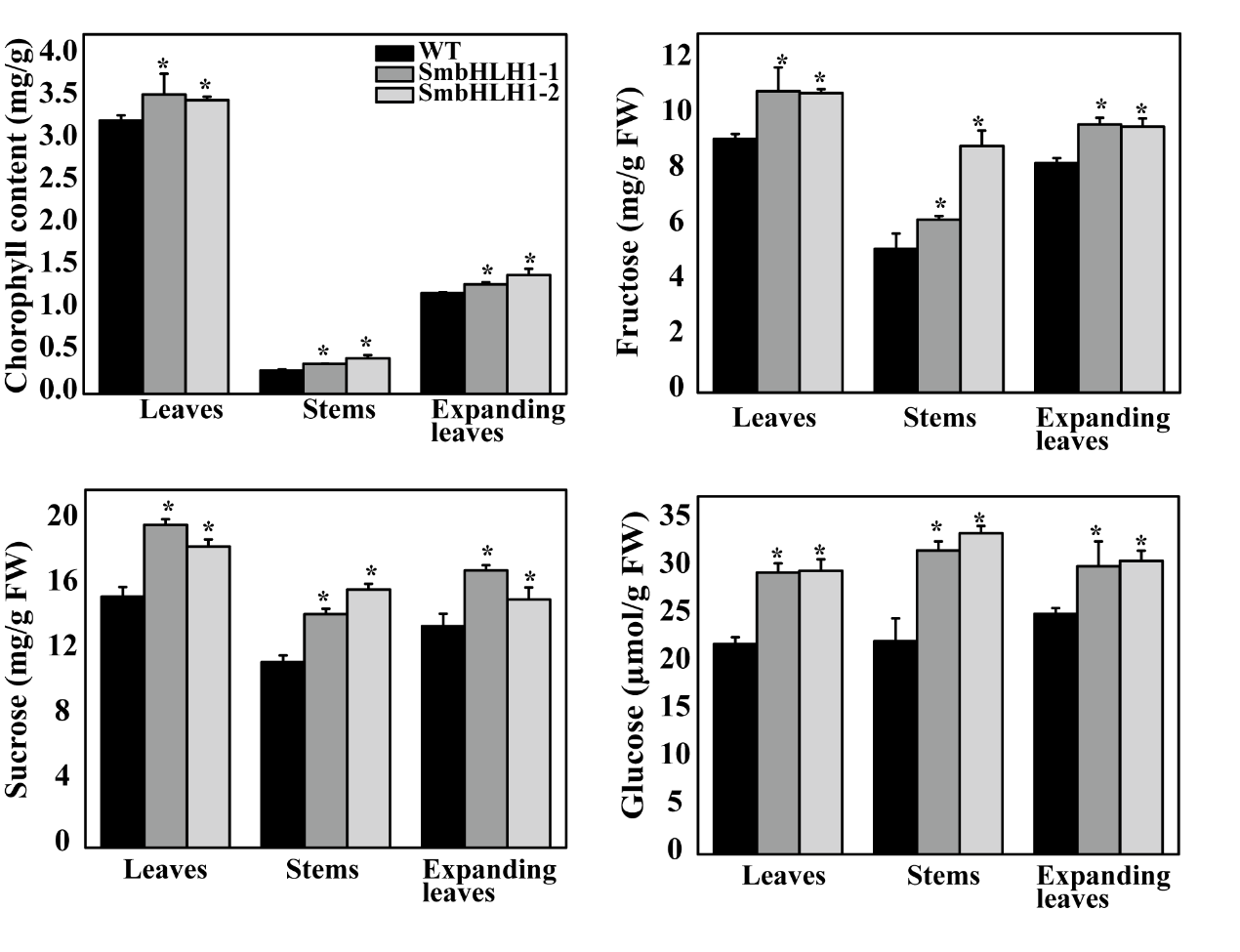


**Supplementary Figure 5.** *SmbHLH1*-over-expression alters chlorophyll, glucose, fructose and sucrose content in the eggplant stems. Bars represent means ± SD of three biological replicates. Duncan’s Multiple Range test (*P< 0.05) was used to analyze statistical significance.

**Supplementary Table 1 List of primer sequences used in this study.**

| **Gene** | **Forward primer (5’-3’)** | **Reverse primer (5’-3’)** |
| --- | --- | --- |
| **For cDNA sequence** **isolation** | | |
| *SmbHLH1* | ATGCAGGCCCAGATCCATGG | TCAAGGAGATTCAAGCCCACTGC |
| *SmTT8* | ATGATGGAGATCATACAGCCTAGCA | AACTCTAGGGATTATCTGATGTATTGA |
| **For RT-qPCR** | | |
| *Smactin* | GTCGGAATGGGACAGAAGGATG | GTGCCTCAGTCAGGAGAACAGGGT |
| *SmCHS* | GGGAACAGTACTCCGGCTAGCC | AACACCTGAAATTGGGTCTGAACCA |
| *SmCHI* | CCTTGACGGGTAAGCAATACTCTG | GATGGAGGCACCATGTGGGAAGG |
| *SmF3H* | GTGGTCCAAGACTGGCGTGAAAT | TTCTCTAACCCCATTGCTTCTGATA |
| *SmF3’5’H* | TGGACCTCGTTGGAAGTTGCTAAG | TGCCATCGCGAACGTCAACATAT |
| *SmDFR* | GGCCATTGAGACTTGCCGACAG | CACCATTGGTCAACTGTCCTGTACT |
| *SmANS* | CTCGATTCCCACCTCGGACCTT | TCAGCTGCAGCGTCCTGTTTGT |
| *SmbHLH1* | TGATCCTGAATCCACCGAATGT | TTGCGCCCACTATCCATATGTG |
| *SmTT8* | TTCTGCCGGAGACTCAAATCAG | AAACCATTCGGATTCCGTCAAG |
| **For subcellular localization analysis** | | |
| *SmbHLH1* | TCTCTCTCTCAAGCTTATGCAGGCCCAGATCCATGG | TGCTCACCATGGATCCAGGAGATTCAAGCCCACTGC |
| **For plant transformation** | | |
| *SmbHLH1* | CGGTCGACATGCAGGCCCAGATCCATGG | CGGGATCCTCAAGGAGATTCAAGCCCACTGC |
| **For Y2H and BiFC assay** | | |
| *pGBKT7-SmTT8* | AGGAGGACCTGCATATGATGATGGAGATCATACAGCCTAGCA | GGCCGCTGCAGGTCGACAACTCTAGGGATTATCTGATGTATTGA |
| *pGBKT7-SmbHLH1* | AGGAGGACCTGCATATGATGCAGGCCCAGATCCATGG | GGCCGCTGCAGGTCGACAGGAGATTCAAGCCCACTGC |
| *pGADT7-SmMYB113* | GGAGGCCAGTGAATTCATGAATAATCCTCCTATAATCTGTACGTC | ATGGATCCCGTATCGATATCAAGTAGATTCCATAAATCAATATCA |
| *pXY104-SmMYB113* | AGGTACCCGGGGATCCATGAATAATCCTCCTATAATCTGTACGTC | TGCCACCGCCGTCGACATCAAGTAGATTCCATAAATCAATATCA |
| *pXY106-SmbHLH1* | GGACGCCGGCGGATCCATGCAGGCCCAGATCCATGG | AGCTCTGCAGGTCGACAGGAGATTCAAGCCCACTGC |
| *pXY106-SmTT8* | GGACGCCGGCGGATCCATGATGGAGATCATACAGCCTAGCA | AGCTCTGCAGGTCGACAACTCTAGGGATTATCTGATGTATTGA |
| **For Y1H assay** | | |
| *pLacZi-proSmCHS* | CCGGGGATCTGTCGACCAACTGCATTCATTGTAACTAAATGTCA | GAGCACATGCCTCGAGTTTCGCCCGAAAAAATGGT |
| *pLacZi-proSmCHI* | CCGGGGATCTGTCGACCATGCCAAGTCAAAATAGGATTAGC | GAGCACATGCCTCGAGTGAAATCTTCTGGTATTACACTCTT |
| *pLacZi-proSmF3H* | CCGGGGATCTGTCGACTCTAGCTCAATTTTAAAACAACGG | GAGCACATGCCTCGAGTTTAACTTTTTCTTGTTTTTGGTT |
| *pLacZi-proSmF3’5’H* | CCGGGGATCTGTCGACAAAAAAGTTGACTAAAATGGAAAAC | GAGCACATGCCTCGAGCCCACAAGTGCCAACTTT |
| *pLacZi-proSmDFR* | CCGGGGATCTGTCGACACATGATTTACATCATAATTGTAGGC | GAGCACATGCCTCGAGTTTCAGAAATGTAAGGTAAAAAGAG |
| *pLacZi-proSmANS* | CCGGGGATCTGTCGACACGTGGTGTTCAATAACTCCA | GAGCACATGCCTCGAGCTCTTTAACGCGGAGTACTT |
| *pB42AD-SmbHLH1* | TGCCTCTCCCGAATTCATGCAGGCCCAGATCCATGG | TCCAAAGCTTCTCGAGAGGAGATTCAAGCCCACTGC |
| **For dual-luciferase assay** | | |
| *proSmDFR* | CGGTATCGATAAGCTTACATGATTTACATCATAATTGTAGGC | TAGAACTAGTGGATCCTTTCAGAAATGTAAGGTAAAAAGAG |
| *proSmANS* | CGGTATCGATAAGCTTACGTGGTGTTCAATAACTCCA | TAGAACTAGTGGATCCCTCTTTAACGCGGAGTACTT |
| *pHB-SmMYB113* | TCTCTCTCTCAAGCTTATGAATAATCCTCCTATAATCTGTACGTC | TGCAGCTCGAGGATCCTTAATCAAGTAGATTCCATAAATCAATATCA |
| *pHB-SmbHLH1* | TCTCTCTCTCAAGCTTATGCAGGCCCAGATCCATGG | TGCAGCTCGAGGATCCTCAAGGAGATTCAAGCCCACTGC |
| *pHB-SmTT8* | TCTCTCTCTCAAGCTTATGATGGAGATCATACAGCCTAGCA | TGCAGCTCGAGGATCCTTAAACTCTAGGGATTATCTGATGTATTGA |
